# The Mutual Support Model of Mindfulness and Character Strengths

**DOI:** 10.1007/s12671-019-01103-z

**Published:** 2019-02-16

**Authors:** Dandan Pang, Willibald Ruch

**Affiliations:** Section of Personality and Assessment, Department of Psychology, University of Zurich, Binzmuehlestrasse 14, 8050 Zurich, Switzerland

**Keywords:** Character strengths, Mindfulness, MBSR, Positive psychology, VIA classification

## Abstract

**Objectives:**

Numerous studies have confirmed robust relationships between general well-being and mindfulness or character strengths, respectively, but few have examined associations between mindfulness and character strengths. Two studies were carried out to explore these relationships comprehensively in the framework of the Values in Action (VIA) classification of character strengths.

**Methods:**

In study 1, participants (*N* = 1335) completed validated assessments of mindfulness and character strengths, and the relationship between the two was investigated in a broad online sample. In study 2, the effect of a mindfulness training on specific character strengths was investigated using a randomized-control design (*N* = 42).

**Results:**

The results of study 1 confirmed positive relationships between mindfulness and character strengths and further identified a list of character strengths that might overlap with mindfulness—i.e., creativity, curiosity, open-mindedness, love of learning, perspective, bravery, perseverance, zest, love, social intelligence, forgiveness, self-regulation, appreciation of beauty, gratitude, hope, and spirituality. The findings of study 2 provided further support for the hypothesis that mindfulness training could help cultivate certain character strengths. Compared with participants in the waitlist control condition, those who attended an 8-week mindfulness-based training program showed significant increases in the strengths of love, appreciation of beauty, gratitude, and spirituality, and a trend toward significant increases in the strengths of zest and bravery.

**Conclusions:**

The results provide initial evidence for a mutual support model of mindfulness and character strengths.

Mindfulness describes a particular way of paying attention to the present moment without judgment (Kabat-Zinn 1994). In less than 40 years, it has become a booming area of scientific research in psychology. In recent years, there has been a spate of particular interest in implementing mindfulness in the specific context of positive psychology (e.g., Baer 2015; Baer and Lykins 2011; Ivtzan and Lomas 2016; Malinowski 2013). Known as the science of well-being, positive psychology (Seligman and Csikszentmihalyi 2000) focuses on promoting human potential (Sheldon and King 2001). Its central tenet is that mental health is more than the absence of pathology or distress (as already noted by Marie Jahoda in 1958); therefore, psychological science should also investigate how individuals and communities can flourish and thrive (Peterson 2006).

As one of the original “three pillars” of positive psychology (Seligman 2002), character strengths, together with virtues, have developed into a fast-growing research topic in psychology during the past decade. Character strengths are a family of positive personality traits that are morally and positively valued but have been neglected within personality psychology despite empirical overlaps between character and personality traits, such as agreeableness and conscientiousness (Macdonald et al. 2008). Peterson and Seligman (2004) proposed a comprehensive system of positive traits, labeled the Values in Action (VIA) classification of character strengths. These efforts represented “new attention paid to adaptive, constructive and growth-oriented aspects of personality” (McCrae 2011, p. 196). As one of the most comprehensive structure of character, the VIA classification identified 24 character strengths and categorized them into six virtues, which were considered universal across time and different cultures (e.g., Dahlsgaard et al. 2005).

Existing research on the overlap between personality traits and dispositional mindfulness or mindfulness meditation has primarily focused on either the five-factor model of personality or “Big 5” (McCrae and Costa 1987; McCrae and John 1992) or the “psychobiological” model of personality with an emphasis on the character profile (Cloninger et al. 1993). For example, a recent meta-analysis synthesized findings from 32 samples in 29 studies and confirmed that trait mindfulness correlated negatively with neuroticism but positively with conscientiousness (Giluk 2009). A cross-sectional study (van den Hurk et al. 2011) revealed that mindfulness meditators showed higher scores of openness but lower scores of conscientiousness than non-meditators. They also found that the practice of mindfulness meditation was negatively related to neuroticism and positively related to openness and extraversion.

The psychobiological model of personality consists of three dimensions that constitute the character profile: (1) self-directedness, which maps onto concepts such as self-esteem and self-efficacy; (2) cooperativeness, which expresses the capacity to be empathic, tolerant, and compassionate; and (3) self-transcendence, which measures the tendency toward spirituality and creativeness (Crescentini and Capurso 2015). A variety of studies have investigated the relationships between mindfulness and positive traits, such as the character component of the psychobiological model of personality. For example, after an eight-week program of mindfulness meditation, participants in an experimental group scored higher in all three aspects of the character profile, while no changes were found in the control group (Campanella et al. 2014). Advanced meditators who had more than two years of meditation experience scored higher in all three aspects of the character profile compared with naïve subjects (Crescentini and Capurso 2015; Haimerl and Valentine 2001).

Much less is known about the overlap between mindfulness and the VIA classification of character strengths, although numerous studies have independently demonstrated the benefits of mindfulness and character strengths (e.g., Grossman et al. 2004; Sin and Lyubomirsky 2009). Given the empirical overlap of VIA character strengths with the Big 5 (e.g., Macdonald et al. 2008), as well as the conceptual connections with the character profile (Crescentini and Capurso 2015), it is natural to assume that mindfulness and character strengths (as viewed within the VIA framework) are intimately associated. The first link between mindfulness and character strengths is the similarity in their functions. Peterson and Seligman (2004) described how character strengths can contribute to a more fulfilling life, which was in accordance with the general idea behind mindfulness in the Buddhist tradition, in which the Buddha also searched for meaning and happiness (Garfinkel 2007). This association has been confirmed by extant evidence on the robust relationships between mindfulness and character strengths with well-being (for overviews, see e.g., Bruna et al. 2018; Eberth and Sedlmeier 2012).

Second, a closer look at the definitions of the two constructs also unveils similarities. Researchers had devoted to establishing a consensus on the conceptualization of mindfulness and eventually came up with a mutually agreed operational definition: “mindfulness involves the *self-regulation* of attention with an approach of *curiosity*, *openness* and *acceptance*” (Bishop et al. 2004). In this definition, one could easily relate the character strengths of curiosity, open-mindedness, and self-regulation to mindfulness.

Third, this overlap is apparent regarding the nature of how people practice and master mindfulness. The idea that mindfulness can be cultivated through meditation exercises (Hanh 1975; Kabat-Zinn 1990, 1994; Linehan 1993), especially Buddhist-based meditations, is an essential part of Eastern philosophies (Feuerstein 2001). Many mindfulness meditations have a wisdom component, such as promoting a “wise mind” (Linehan 1993) and “wisdom meditation” (Kristeller 2003). Therefore, positive correlations can be expected to exist between mindfulness and character strengths assigned to the virtue of wisdom (creativity, curiosity, love of learning, open-mindedness, and perspective). Since several mindfulness-based programs (such as mindfulness-based stress reduction [MBSR]; Kabat-Zinn 1982) have helped patients with chronic pain, the strengths of bravery, perseverance, and self-regulation could also be related to mindfulness. Mindfulness exercises require us to keep our attention alive to the present moment (Hanh 1975), which means keeping enthusiasm and energy for the here and now. This in turn leads to a positive association between mindfulness meditations and zest. The observing component of mindfulness emphasizes the importance of observing, noticing, or attending to a variety of stimuli, which is also critical for the strength of appreciation of beauty.

Despite the theoretical linkage between mindfulness meditations and character strengths, few empirical studies have investigated their interconnections. Their results were considered piecemeal (e.g., studies focused on one strength), indirect, or non-inclusive (Niemiec 2014). According to Baer and Lykins’ (2011) summary, mindfulness (e.g., mindfulness-based intervention) was associated with increased curiosity, openness to experience, vitality, emotional intelligence (related to social intelligence), self-regulation, optimism/hope, and states of transcendence (especially spirituality). However, they also pointed out the need for additional empirical examinations of these relationships. Two recent studies (Duan 2016; Duan and Ho 2018) showed that two components of dispositional mindfulness (observing and non-judging) were related to individual strengths. Using the Brief Strength Scale (Ho et al. 2016), which categorized strengths into three types (interpersonal, intellectual, and temperance strengths), the authors provided an overview of the relationships between facets of dispositional mindfulness and strengths. However, the mechanisms depicted in the studies could be mixed since the strengths were grouped. To capture the relationship with full pictures of individual strengths, more comprehensive measures and variant samples are required.

In sum, the two studies presented here attempt to derive, both theoretically and empirically (from preliminarily results), a list of character strengths that could potentially relate to mindfulness: creativity, curiosity, open-mindedness, love of learning, perspective, bravery, perseverance, zest, social intelligence, self-regulation, appreciation of beauty, hope, and spirituality. To date, no study has examined the relationships between mindfulness and character strengths within the VIA classification framework. A mutual support model of mindfulness and character strengths is proposed and initially tested in the present study. It is assumed that the link between mindfulness and character strengths will be bidirectional. That is, certain character strengths will facilitate the practice of mindfulness, while mindfulness through practice will have an impact on the cultivation of certain character strengths.

## Study 1

The aim of study 1 was to investigate the overlap between mindfulness and character strengths using comprehensive measures and a broad sample. First, it was hypothesized that certain character strengths as mentioned in the introduction (i.e., creativity, curiosity, open-mindedness, love of learning, perspective, bravery, perseverance, zest, social intelligence, self-regulation, appreciation of beauty, hope, and spirituality) would correlate with the five facets and the total score of mindfulness. Second, it was hypothesized that participants currently practicing mindfulness meditation would score higher in those character strengths compared with participants with no mindfulness experience.

## Method

### Participants

A total of 1471 participants completed a set of online questionnaires on mindfulness and character strengths. A preliminary analysis resulted in the removal of the data from 136 participants for the following reasons: (1) seven participants rated at least 80% of the questionnaires with the same value; (2) thirty-seven participants claimed to have no meditation experience but at the same time reported themselves as practicing meditation regularly or irregularly; (3) ninety-two meditators reported that they did not practice Buddhist-based meditation or Christian practices (e.g., yoga, tai chi, or prayer), or did not specify their meditation type. The final sample consisted 1335 German-speaking volunteers (349 men, 986 women) aged between 18 and 79 years (*M* = 42.5, SD = 12.0). Most participants were from Germany (65%), with smaller numbers from Switzerland (23.7%) and Austria (8.5%). More than half of the participants had a university degree or were currently studying (63.7%). In addition, participants’ experience of meditation was measured following the procedure adapted from Baer et al. (2008). They were asked if they had any previous meditation experience before with three possible responses: (1) Yes, I currently meditate; (2) Yes, but it was a while ago; or (3) No, I do not have any experiences with meditation. Based on their answers, participants were split into two different groups: (1) the current meditators (i.e., those who selected the first option; *n* = 437) and (2) the non-meditators (i.e., those who selected the third option; *n* = 429). The two samples differed significantly in their age, *t*(1, 864) = 9.00, *p* < .001, but the proportion of men and women (*χ*^2^[1] = 1.36, *p* = .243) and their education level (*χ*^2^[4] = 4.89, *p* = .298) did not differ between the two groups.

### Procedures

Participants were requested to complete the questionnaires on a website (www.charakterstaerken.org; hosted by the Section on Personality and Assessment of the University of Zurich) for research purposes between May 2015 and February 2017. The website was promoted by various means to obtain a heterogeneous sample; these included press coverages, publishing the link online, and contacting specific groups. The volunteers registered on the website from their personal computers and completed the questionnaires online. Respondents were not paid for participating but were provided with an automatically generated feedback of their individual results. The procedure was conducted in accordance with the guidelines of the Ethics Committee of the Department of Psychology at the University of Zurich.

### Measures

#### Mindfulness

The Five Facet Mindfulness Questionnaire (FFMQ; Baer et al. 2006) is a self-report instrument consisting of 39 items. Respondents use a 5-point scale to rate their dispositional mindfulness with five facets: observing, describing, acting with awareness, non-judging of experience, and non-reacting. A sample item for the facet of describing is: “I’m good at finding words to describe my feelings.” In the present study, the German version of the questionnaire was used (FFMQ-D; Michalak et al. 2016). Satisfactory internal consistencies were found for all three samples and all five facets. Cronbach’s *α* ranged from .76 to .92 (median = .87).

#### Character Strengths

The Values in Action Inventory of Strengths (VIA-IS; Peterson et al. 2005) is a self-report questionnaire consisting of 240 items that measure the 24 character strengths of the VIA classification. A sample item for the strength of perseverance is: “I never quit a task before it is done.” In the current study, the German version of the VIA-IS was used (Ruch et al. 2010), which showed high reliability across all samples. Cronbach’s *α* ranged from .71 to .89 (median = .79).

### Data Analyses

First, Spearman’s rank correlation was conducted between the five facets as well as the total score of mindfulness and the 24 character strengths because several facets of mindfulness (i.e., observing, describing, and non-judging) and several scales of character strengths (e.g., curiosity) were excessively skewed. Age, gender, and education were controlled to partial out the minor sources of variance within the sample, although doing so did not alter the findings.

An independent samples *t* test was conducted to explore the differences in mindfulness and character strengths between the current meditators and the non-meditators. Because the two samples differed significantly in age (those who are older are more likely to have a longer experience of mindfulness practice), a case-control match using SPSS software was conducted before further comparisons (matching variable: age; tolerance value, 1). After matching, two samples that no longer differed in age were obtained: (1) the current meditators (*n* = 316, *M*_age_ = 42.9) and (2) the non-meditators (*n* = 316, *M*_age_ = 43.2). Subsequently, standardized effect sizes were calculated using Cohen’s *d* (Cohen 1988).

## Results

The results of the descriptive statistics and the correlations between the five facets as well as the total score of mindfulness and the 24 character strengths are displayed in Table 1. As shown in Table 1, almost all mindfulness facets and the total score of mindfulness correlated positively with the character strengths when demographics (age, gender, and education) were controlled. The character strengths of hope, bravery, curiosity, social intelligence, zest, love, perspective, gratitude, self-regulation, and creativity displayed medium effect correlations with at least one facet of mindfulness and the total score of mindfulness. In contrast, modesty and prudence were either negatively correlated or unrelated to mindfulness. In addition, despite a lower correlation with the total score of mindfulness, forgiveness (*r*_non-reacting_ = .32, *p* < .001), perseverance (*r*_awareness_ = .33, *p* < .001), open-mindedness (*r*_describing_ = .30, *p* < .001), and appreciation of beauty (*r*_observing_ = .46, *p* < .001) correlated positively with one facet of mindfulness but not the remaining facets. All *p* values were corrected using the Bonferroni method.

**Table 1:** Descriptive statistics and correlations between mindfulness and the 24 character strengths controlled for age, gender, and education

			FFMQ					
VIA-IS	*M*	SD	OB	DS	AW	NJ	NR	TOT-M
Hope	3.54	0.61	.24^***^	.28^***^	*.34* ^***^	*.35* ^***^	*.45* ^***^	*.48* ^***^
Bravery	3.60	0.53	*.32* ^***^	*.41* ^***^	*.33* ^***^	.27^***^	*.38* ^***^	*.48* ^***^
Curiosity	4.01	0.52	*.36* ^***^	.29^***^	.25^***^	.31^***^	*.37* ^***^	*.45* ^***^
Social intelligence	3.77	0.49	*.35* ^***^	*.45* ^***^	.29^***^	.18^***^	*.33* ^***^	*.44* ^***^
Zest	3.51	0.59	.27^***^	.27^***^	*.33* ^***^	*.31* ^***^	*.37* ^***^	*.44* ^***^
Love	3.88	0.55	.26^***^	*.38* ^***^	.26^***^	.24^***^	.26^***^	*.39* ^***^
Perspective	3.63	0.49	.23^***^	*.35* ^***^	.28^***^	.23^***^	*.32* ^***^	*.39* ^***^
Gratitude	3.84	0.55	*.40* ^***^	.23^***^	.22^***^	.18^***^	.29^***^	*.36* ^***^
Self-regulation	3.28	0.57	.20^***^	.17^***^	.35^***^	.18^***^	*.31* ^***^	*.33* ^***^
Creativity	3.59	0.67	*.34* ^***^	.25^***^	.13^***^	.16^***^	.23^***^	*.30* ^***^
Humor	3.52	0.63	.23^***^	.18^***^	.16^***^	.17^***^	.29^***^	.29^***^
Love of learning	3.94	0.58	.29^***^	.22^***^	.13^***^	.17^***^	.24^***^	.29^***^
Forgiveness	3.58	0.55	.15^***^	.12^***^	.18^***^	.22^***^	*.32* ^***^	.28^***^
Leadership	3.72	0.50	.18^***^	.24^***^	.17^***^	.15^***^	.27^***^	.28^***^
Spirituality	3.12	0.93	.27^***^	.19^***^	.16^***^	.14^***^	.23^***^	.28^***^
Perseverance	3.51	0.63	.14^***^	.21^***^	*.33* ^***^	.12^***^	.20^***^	.27^***^
Open-mindedness	3.94	0.49	.21^***^	*.30* ^***^	.19^***^	.07	.19^***^	.26^***^
Appreciation beauty	3.61	0.57	*.46* ^***^	.16^***^	.03	.03	.12^***^	.21^***^
Honesty	3.85	0.44	.14^***^	.13^***^	.21^***^	.11^***^	.13^***^	.19^***^
Kindness	3.83	0.47	.23^***^	.18^***^	.10^***^	.04	.12^***^	.18^***^
Fairness	3.96	0.45	.15^***^	.09	.12^***^	.09	.16^***^	.16^***^
Teamwork	3.62	0.50	.07	.08	.10^***^	.09	.16^***^	.14^***^
Prudence	3.45	0.56	.08	.09	.12^***^	− .01	.09	.09
Modesty	3.23	0.56	− .06	− .20^***^	− .02	− .05	− .01	− .10^***^
*M*			3.69	3.78	3.36	3.64	3.15	3.52
SD			0.60	0.74	0.66	0.85	0.71	0.51

*N* = 1335. *M*, mean; *SD*, standard deviation. *VIA-IS*, Values in Action Inventory of Strengths. *FFMQ*, Five Facet Mindfulness Questionnaire. *Appreciation beauty*, appreciation of beauty and excellence. *OB*, observing; *DS*, describing; *AW*, awareness; *NJ*, non-judging; *NR*, non-reacting; *TOT-M*, total score of mindfulness. Age, gender, and education were controlled to partial out the minor sources of variance within the sample, although doing so did not alter the findings. The order is sorted by the size of correlations with the total score of mindfulness. Correlations that were equal or larger than .30 are in italics. Results with three asterisks (^***^) indicate statistical significance using the Bonferroni corrections (*p* < .0003) for multiple comparisons (144 tests)

Next, an independent samples *t* test was conducted to investigate the differences between the current meditators and the non-meditators (after matching their age) regarding their mindfulness level and character strengths. The results are displayed in Table 2. Significant differences were found between the two matched samples for all five facets of mindfulness and certain character strengths. As shown in Table 2, spirituality showed a large effect size, while gratitude, appreciation of beauty, curiosity, and love of learning displayed medium effect sizes, indicating that the current meditators scored higher on those character strengths than the non-meditators. Despite not reaching statistical significance after Bonferroni corrections, the current meditators showed a tendency to score higher in strengths of leadership, zest, perspective, self-regulation, and humor. In contrast, the strengths of kindness, perseverance, fairness, open-mindedness, teamwork, and prudence showed no difference between the two groups, while strengths of honesty and modesty showed a tendency in the opposite direction.

**Table 2:** Mean differences of mindfulness and character strengths between the current meditators and the non-meditators after matching age

	Current meditators		Non-meditators		Difference		Effect size
Measures	*M*	SD	*M*	SD	*t* (630)	*p*	Cohen’s *d*
FFMQ							
Observing	3.93	0.52	3.46	0.62	10.33	< .001^a^	0.82
Non-reacting	3.40	0.69	2.94	0.68	8.28	< .001^a^	0.66
Describing	4.01	0.66	3.65	0.78	6.32	< .001^a^	0.51
Non-judging	3.88	0.81	3.51	0.84	5.72	< .001^a^	0.46
Awareness	3.51	0.66	3.27	0.65	4.60	< .001^a^	0.37
TOT-M	3.75	0.48	3.37	0.48	9.90	< .001^a^	0.79
VIA-IS							
Spirituality	3.66	0.82	2.69	0.89	14.24	< .001^a^	1.13
Gratitude	4.01	0.53	3.67	0.56	7.85	< .001^a^	0.62
Appreciation beauty	3.75	0.52	3.44	0.59	6.95	< .001^a^	0.56
Love of learning	4.08	0.53	3.77	0.63	6.62	< .001^a^	0.53
Curiosity	4.16	0.47	3.9	0.56	6.37	< .001^a^	0.50
Forgiveness	3.68	0.53	3.46	0.55	5.09	< .001^a^	0.41
Love	3.99	0.53	3.78	0.59	4.55	< .001^a^	0.38
Creativity	3.71	0.67	3.47	0.72	4.30	< .001^a^	0.35
Hope	3.66	0.59	3.47	0.65	3.86	< .001^a^	0.31
Bravery	3.71	0.52	3.55	0.56	3.72	< .001^a^	0.30
Social intelligence	3.85	0.47	3.70	0.52	3.73	< .001^a^	0.30
Leadership	3.78	0.50	3.64	0.51	3.37	.001^a^	0.28
Zest	3.60	0.59	3.45	0.60	3.13	.002	0.25
Perspective	3.71	0.48	3.60	0.52	2.76	.006	0.22
Self-regulation	3.36	0.58	3.24	0.59	2.60	.010	0.21
Humor	3.58	0.64	3.46	0.67	2.27	.024	0.18
Kindness	3.86	0.47	3.80	0.51	1.51	.131	0.12
Perseverance	3.51	0.65	3.55	0.64	− 0.87	.386	0.06
Fairness	3.95	0.45	3.93	0.43	0.71	.478	0.05
Open-mindedness	3.95	0.47	3.93	0.51	0.49	.622	0.04
Teamwork	3.59	0.49	3.59	0.50	0.06	.955	0.00
Prudence	3.43	0.59	3.44	0.57	− 0.19	.853	− 0.02
Honesty	3.82	0.44	3.91	0.43	− 2.51	.012	− 0.21
Modesty	3.15	0.57	3.27	0.59	− 2.60	.010	− 0.21

*n* = 316 for each group. *M*, mean; *SD*, standard deviation. *TOT-M*, total score of mindfulness. Appreciation beauty, appreciation of beauty and excellence. For all measures, higher means indicate higher scores. The order is sorted by the effect size Cohen’s *d*. ^a^Results with the superscript indicate statistical significance using the Bonferroni corrections (*p* < .0017) for multiple comparisons (30 tests)

## Discussion

**Fig. 1:**
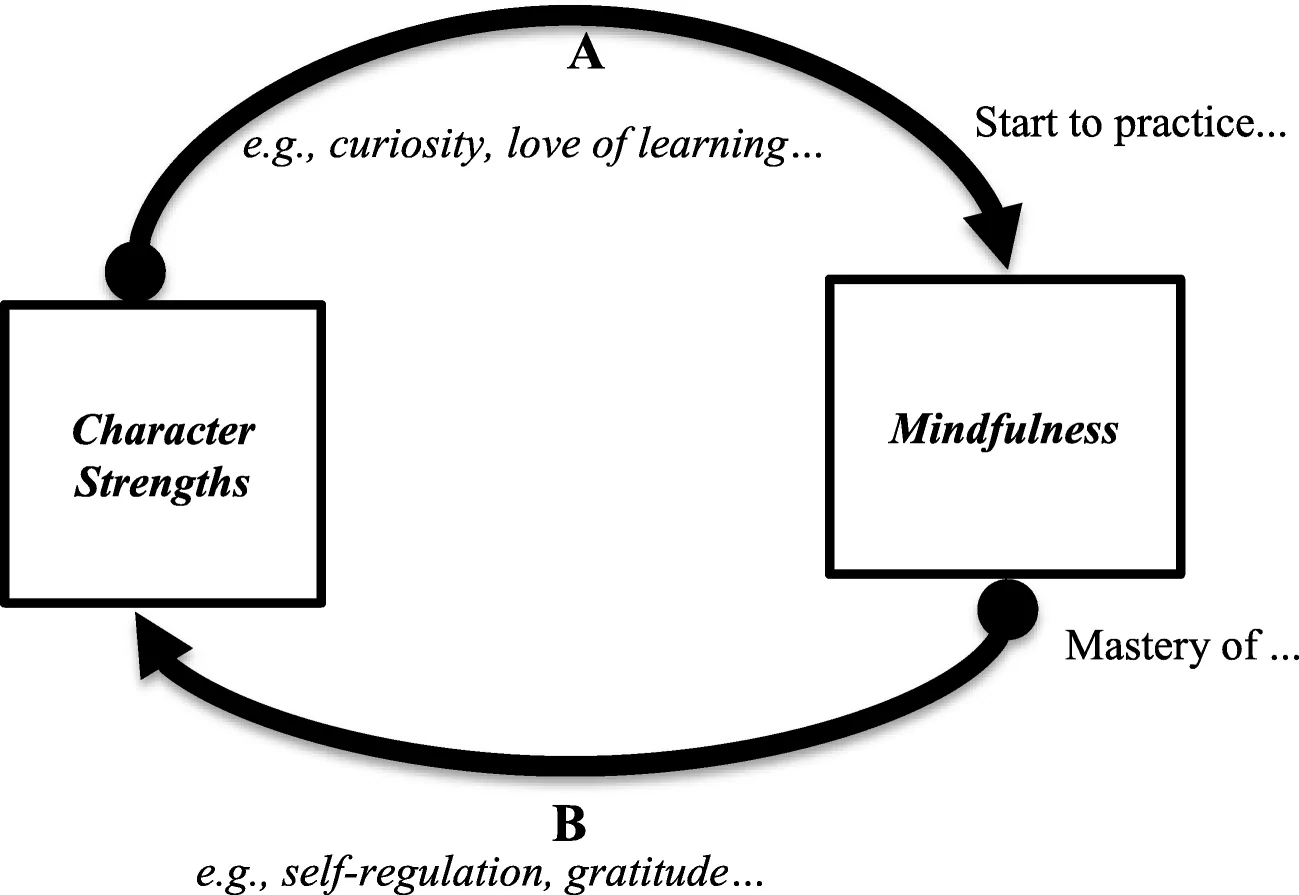
The proposed mutual support model of mindfulness and character strengths

Combining the results of Table 1 and Table 2, a list of character strengths that were considered to be overlapping with mindfulness and its facets was derived: creativity, curiosity, open-mindedness, love of learning, perspective, bravery, perseverance, zest, love, social intelligence, forgiveness, self-regulation, appreciation of beauty, gratitude, hope, and spirituality. These were the strengths that correlated with mindfulness or at least one facet of mindfulness with medium to large effect sizes or were notably different between the current meditators and the non-meditators (with medium to large effect sizes). Based on these results, as well as the theoretical connections between the two as mentioned in the introduction, a mutual support model of mindfulness and character strengths (Fig. 1) was proposed. The model assumes that certain character strengths (e.g., curiosity) facilitate mindfulness; i.e., people with these character strengths are more willing to try mindfulness meditations (path A). Conversely, the mastery of mindfulness is assumed to enhance certain character strengths, such as spirituality (path B). However, because of the nature of a cross-sectional study, no causality or direction could be derived from the current results, i.e., which character strengths belong to path A, and which belong to path B. Therefore, an intervention study with a control group was conducted to find out exactly which character strengths might be enhanced through a mindfulness training (path B).

## Study 2

Study 1 produced a list of character strengths that seemed to overlap with mindfulness and resulted in the proposal of a mutual support model of mindfulness and character strengths (see Fig. 1). However, the model could not be tested with a cross-sectional design. In study 2, the aim was to test whether character strengths suggested by study 1 could be enhanced through a mindfulness training, and therefore evidence path B in the mutual support model.

## Method

### Participants

To be eligible to take part, participants in the study had to meet the following criteria: (a) they were adults aged 18 years or older; (b) they had no previous meditation experience; (c) their level of employment ≥ 50%; and (d) they were neither attending psychotherapeutic treatment nor using psychotropic/illegal drugs throughout the duration of the study. Eighty-six volunteers signed up for participation in the study through a web link via the Unipark platform, where they completed a screening and their demographic details. A total number of 63 participants from various areas of employment were randomly assigned to three different conditions: (1) mindfulness-based strengths practice (MBSP; Niemiec 2013; *n* = 21); (2) mindfulness-based stress reduction (MBSR; Kabat-Zinn 1982; *n* = 21); and (3) waitlist control condition (WL; *n* = 21). In the present study, we focused on the participants of two conditions: MBSR vs. WL to answer our specific research question*.* The randomization was constrained because of the limited availability of some participants; their group was adjusted accordingly. However, this would not impact our randomization because the participants did not know to which conditions they were assigned. They were all informed that they would be participating in a mindfulness-based training without knowing the details of the training.

### Procedure

To promote the study, e-mails were sent to potential target groups, such as HR professionals; the e-mails also included instructions on how to participate. In addition, the study was advertised by various means through the Internet, such as online forums and social media platforms, as well as different mailing lists. To motivate participants and reduce dropout, all participants were asked to pay 100 CHF to attend the interventions and were given individual feedback as an incentive. The procedure was approved by the Ethics Committee of the Department of Psychology at the University of Zurich.

After registration and filling out the baseline measures, participants in the experimental condition gathered once a week for eight consecutive weeks and received a two-hour version of the standard MBSR training, without the retreat that is proposed in the manual of the MBSR curriculum. The trainer was a qualified MBSR teacher who had more than two years of experience in leading MBSR group at the time of the intervention. Participants in the MBSR conditions were asked to complete homework between each session. This consisted of a 20–40-minute session on a daily basis, which required them to repeat certain mindfulness practices using handouts and audio tapes. For the control condition, participants were recruited in the same way as the experimental condition, with an invitation to participate in a mindfulness-based training. However, they were later informed that the current program was fully booked, and they would have to wait a year to attend the next intervention. They were asked to fill out the instruments as well as pay the fee, and the role of the wait list control was explained in the process.

**Fig. 2:**
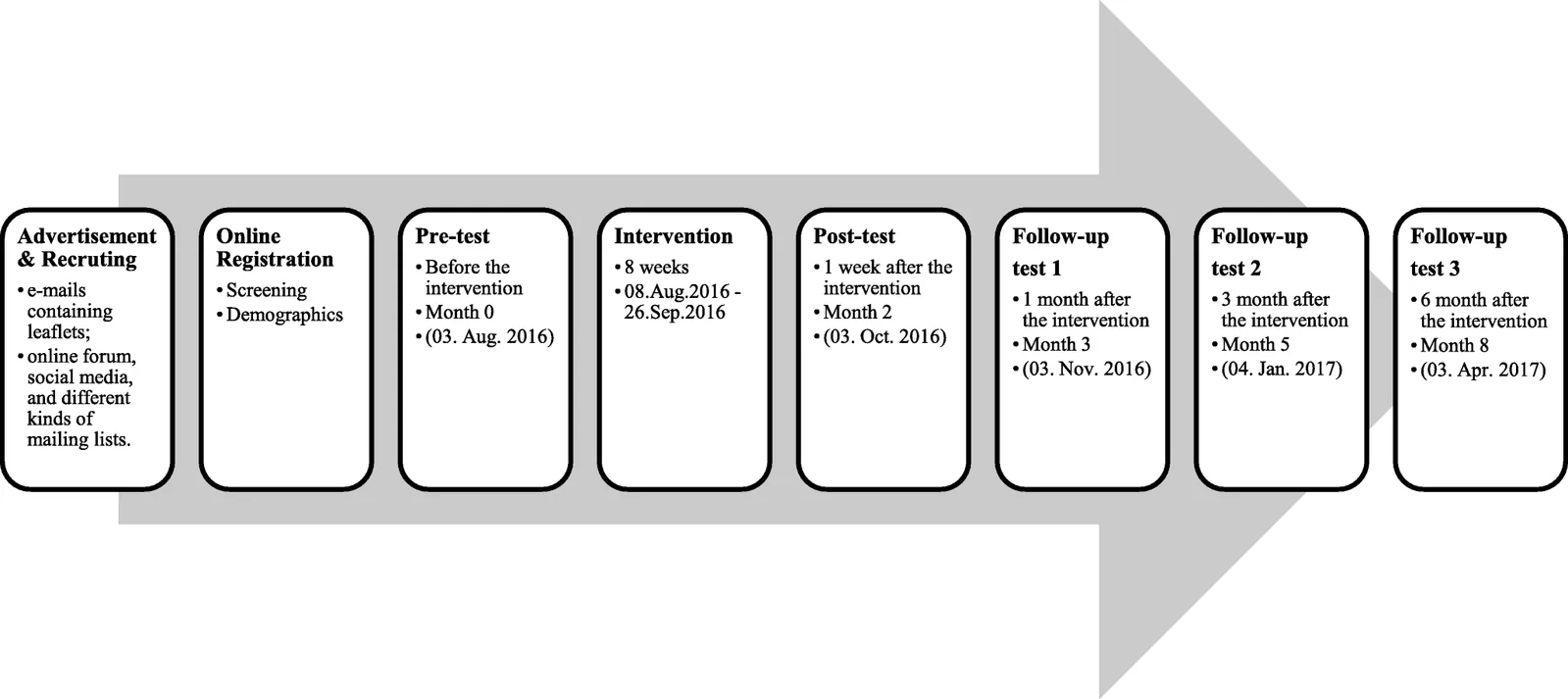
Timeline of the data collection in study 2. Dates in parentheses indicate the points when e-mail reminders to complete the questionnaires were sent to participants

Data were collected online via the Unipark survey platform. E-mail reminders to fill out the questionnaires were sent to participants at the relevant intervals. All participants were asked to complete the same questionnaires at five intermittent points, that is: (1) before the eight-week intervention (month 0); (2) one week after the intervention (month 2); (3) one month after the intervention (month 3); (4) three months after the intervention (month 5); and (5) six months after the intervention (month 8). Figure 2 displays the detailed schedule of the data collection. Participants also reported how often they completed the suggested homework on average on a 6-point scale, both throughout and after the intervention. Data collection lasted through April 2017; the study concluded when participants completed their six-month follow-up assessment.

#### Measures

##### Mindfulness and Character Strengths

The same instruments described in study 1 were used to measure mindfulness and character strengths, namely, the Five Facet Mindfulness Questionnaire (FFMQ; Baer et al. 2006) and the Values in Action Inventory of Strengths (VIA-IS; Peterson et al. 2005).

### Data Analyses

A series of linear mixed effects models were applied, modeling changes over time in participants’ mindfulness (i.e., the total score) and character strengths as suggested by study 1 (i.e., creativity, curiosity, love of learning, perspective, bravery, zest, love, social intelligence, self-regulation, appreciation of beauty, gratitude, hope, and spirituality). The change in the total score of mindfulness serves as an important manipulation check for the MBSR training, and the changes in the character strengths serve as the primary outcomes for the effectiveness of the MBSR. The R package “lme4” (Bates et al. 2015) was used to conduct the analysis, which was based on the restricted maximum likelihood (REML) estimation. The time variable (month) was split into two different phases: (1) from baseline until one week after the intervention (i.e., months 0–2; the acute intervention phase) and (2) from one week after the intervention until the six-month follow-up test (i.e., months 2–8; the follow-up phase). The time variable was dummy coded into two variables: Time1 (0, 2, 2, 2, 2) and Time2 (0, 0, 3, 5, 8) to represent the different time periods. The statistical model can be summarized as follows:

$$ {\displaystyle \begin{array}{l}{Y}_{ij}=\Big[{\gamma}_{00}+{\gamma}_{01}{\mathrm{Condition}}_j+{\gamma}_{10}\mathrm{Time}{1}_{ij}+{\gamma}_{11}{\mathrm{Condition}}_j\ast \mathrm{Time}{1}_{ij}+{\gamma}_{20}\mathrm{Time}{2}_{ij}+\\ {}{\gamma}_{21}{\mathrm{Condition}}_j\ast \mathrm{Time}{2}_{ij}\Big]+\left[{U}_{1j}\ast \mathrm{Time}{1}_{ij}+{U}_{2j}\ast \mathrm{Time}{2}_{ij}+{U}_{0j}+{R}_{ij}\right]\end{array}} $$

Where,

$$ {R}_{ij}\sim N\left(0,{\sigma_R}^2\right)\;\mathrm{and}\;\left\{\begin{array}{l}{U}_{0j}\\ {}{U}_{1j}\\ {}{U}_{2j}\end{array}\right\}\sim N\left\{\begin{array}{l}0\kern0.24em {\tau}_{00}\kern0.24em {\tau}_{01}\kern0.24em {\tau}_{02}\\ {}0,{\tau}_{10}\kern0.24em {\tau}_{11}\kern0.24em {\tau}_{12}\\ {}0\kern0.24em {\tau}_{20}\kern0.24em {\tau}_{21}\kern0.24em {\tau}_{22}\;\end{array}\right\} $$

*Y*_*ij*_ refers to the scores of mindfulness or character strengths at all measurement time points. The training effect was evaluated by examining the Time1*Condition interaction (γ_11_) and Time2*Condition interaction (γ_21_), which reflects group differences in changes from pre-test to post-test and from post-test to follow-up tests, respectively. Missing values were handled by using the multiple imputation (MI) procedure to conduct intent-to-treat analyses. By applying the R package “Amelia” (Honaker et al. 2011), missing data were imputed for each condition at each time point using the expectation maximization (EM) algorithm. This process was repeated 50 times to produce 50 complete datasets where the observed values were the same, and the unobserved values were drawn from their posterior distributions. Effectiveness analyses were then performed on each of the 50 resulting data files, and the 50 estimates were pooled into a single overall estimate using the MI inference rules of “smallsample” (Barnard and Rubin 1999). This method adjusts degrees of freedom for small samples and yields proper *p* values and confidence intervals for the estimates (R package “mice”; Van Buuren and Groothuis-Oudshoorn 2011). Using MI allows a test of whether the same pattern of results would have emerged if dropouts had completed the study.

The effect of mindfulness training on mindfulness and character strengths was evaluated by examining the significant difference between the rates of change (slope) in the scores of character strengths for the experimental condition (MBSR) in comparison with the control condition (WL). That is, the effect was evaluated by examining the Time1*Condition interaction (whether certain character strengths indeed increased after the mindfulness training) and Time2*Condition interaction (whether the increase in certain character strengths changed in the follow-up phase).

## Results

No significant baseline differences were detected across the two conditions for mindfulness and all the character strengths that were suggested by study 1 (*t*(40) ranged from − 1.17 to 0.69, all *p* > .10). Around 80% of the participants were retained at the six-month follow-up test. There were no significant differences based on completion status for the baseline measure, and the dropout rates did not differ across conditions (*χ*^2^(1) = 0.141, *p* = .701). No differences in mindfulness and characters strengths were found between participants who dropped out and those who completed the study (*t*(40) ranged from − 1.06 to 1.90, all *p* > .05). Participants in the intervention condition reported continued engagement with homework throughout the MBSR training and after the MBSR training ended. During the intervention, all participants (100%) reported practicing homework on average once a week or more. When the training was over, still around half of the participants (47.7%) still reported continuing to practice homework once a week or more until six months later.

The results of the descriptive data (means and standard deviations) can be found in Table 3 (using completers’ data), and the results of the piecewise linear mixed effects model are given in Table 4 (using both completers’ and MI data). As shown in Table 4, there were no time effects for all models (after Bonferroni corrections), indicating that participants in the WL condition did not change in their ratings of mindfulness and character strengths over time (both Time1 and Time2), in line with expectations. Only appreciation of beauty and gratitude showed a trend toward a Time1 effect, while love and gratitude showed a trend toward a Time2 effect; this means that caution should be warranted when interpreting the interaction effects on those outcomes.

**Table 3:** Descriptive data of the two conditions at the five time periods for mindfulness and character strengths

		Pre-test			Post-test			1-month FU			3-month FU			6-month FU		
Measures		*n*	*M*	SD	*n*	*M*	SD	*n*	*M*	SD	*n*	*M*	SD	*n*	*M*	SD
FFMQ																
Observing	MBSR	21	3.19	0.67	18	3.74	0.51	18	3.76	0.54	17	3.55	0.59	18	3.72	0.70
	WL	21	3.18	0.63	17	2.81	0.63	16	3.13	0.54	16	3.06	0.70	16	3.20	0.77
Describing	MBSR	21	3.55	0.71	18	3.87	0.49	18	3.75	0.62	17	3.73	0.63	18	3.87	0.52
	WL	21	3.39	0.82	17	3.32	0.80	16	3.35	0.81	16	3.19	1.06	16	3.32	0.99
Awareness	MBSR	21	3.11	0.86	18	3.53	0.56	18	3.49	0.52	17	3.62	0.62	18	3.58	0.54
	WL	21	3.17	0.65	17	3.23	0.59	16	3.29	0.65	16	3.20	0.74	16	3.38	0.76
Non-judging	MBSR	21	3.53	0.94	18	3.87	0.65	18	3.92	0.79	17	3.96	0.76	18	4.04	0.70
	WL	21	3.48	0.86	17	3.74	0.71	16	3.75	0.78	16	3.77	0.85	16	3.84	0.78
Non-reacting	MBSR	21	2.76	0.65	18	3.29	0.60	18	3.35	0.61	17	3.49	0.53	18	3.23	0.54
	WL	21	2.69	0.63	17	2.80	0.54	16	2.88	0.51	16	2.75	0.60	16	2.89	0.65
TOT-M	MBSR	21	3.23	0.51	18	3.66	0.33	18	3.66	0.32	17	3.67	0.36	18	3.69	0.34
	WL	21	3.18	0.46	17	3.18	0.38	16	3.28	0.39	16	3.19	0.56	16	3.32	0.53
VIA-IS																
Creativity	MBSR	21	3.26	0.77	18	3.46	0.55	18	3.50	0.74	17	3.38	0.78	18	3.44	0.76
	WL	21	3.52	0.70	16	3.49	0.64	16	3.57	0.66	16	3.56	0.52	16	3.53	0.61
Curiosity	MBSR	21	3.73	0.53	18	3.94	0.41	18	3.97	0.40	17	3.93	0.37	18	3.91	0.52
	WL	21	3.84	0.61	16	3.71	0.53	16	3.84	0.54	16	3.71	0.49	16	3.71	0.53
Open-mindedness	MBSR	21	3.63	0.55	18	3.82	0.38	18	3.86	0.37	17	3.81	0.38	18	3.88	0.38
	WL	21	3.67	0.54	16	3.66	0.43	16	3.75	0.52	16	3.61	0.56	16	3.76	0.55
Love of learning	MBSR	21	3.45	0.70	18	3.57	0.64	18	3.52	0.61	17	3.59	0.58	18	3.54	0.54
	WL	21	3.60	0.44	16	3.48	0.47	16	3.51	0.43	16	3.53	0.36	16	3.46	0.44
Perspective	MBSR	21	3.42	0.50	18	3.61	0.38	18	3.64	0.44	17	3.56	0.33	18	3.65	0.33
	WL	21	3.40	0.41	16	3.33	0.45	16	3.37	0.46	16	3.34	0.46	16	3.41	0.49
Bravery	MBSR	21	3.41	0.43	18	3.59	0.37	18	3.73	0.41	17	3.61	0.37	18	3.71	0.42
	WL	21	3.39	0.69	16	3.34	0.59	16	3.43	0.64	16	3.44	0.71	16	3.46	0.52
Perseverance	MBSR	21	3.30	0.53	18	3.49	0.45	18	3.41	0.41	17	3.47	0.45	18	3.49	0.40
	WL	21	3.26	0.64	16	3.21	0.64	16	3.34	0.54	16	3.34	0.72	16	3.26	0.61
Zest	MBSR	21	3.32	0.52	18	3.64	0.46	18	3.68	0.45	17	3.52	0.47	18	3.60	0.47
	WL	21	3.46	0.71	16	3.33	0.71	16	3.36	0.69	16	3.24	0.76	16	3.43	0.63
Love	MBSR	21	3.72	0.44	18	3.93	0.39	18	4.03	0.48	17	3.85	0.51	18	3.94	0.52
	WL	21	3.80	0.64	16	3.61	0.63	16	3.66	0.51	16	3.76	0.59	16	3.81	0.57
Social intelligence	MBSR	21	3.65	0.39	18	3.78	0.32	18	3.77	0.31	17	3.74	0.28	18	3.83	0.42
	WL	21	3.57	0.46	16	3.54	0.54	16	3.64	0.48	16	3.66	0.48	16	3.64	0.49
Forgiveness	MBSR	21	3.34	0.49	18	3.47	0.40	18	3.56	0.44	17	3.47	0.42	18	3.63	0.47
	WL	21	3.26	0.46	16	3.18	0.45	16	3.36	0.35	16	3.38	0.37	16	3.38	0.39
Self-regulation	MBSR	21	3.10	0.52	18	3.33	0.49	18	3.42	0.38	17	3.30	0.44	18	3.36	0.48
	WL	21	3.07	0.65	16	3.07	0.41	16	3.14	0.50	16	3.09	0.52	16	3.15	0.45
Appreciation of beauty	MBSR	21	3.36	0.60	18	3.66	0.51	18	3.61	0.48	17	3.61	0.59	18	3.63	0.49
	WL	21	3.42	0.66	16	3.25	0.68	16	3.24	0.61	16	3.31	0.64	16	3.39	0.62
Gratitude	MBSR	21	3.60	0.60	18	3.91	0.57	18	3.94	0.59	17	3.82	0.66	18	3.86	0.71
	WL	21	3.59	0.60	16	3.32	0.47	16	3.41	0.57	16	3.40	0.60	16	3.46	0.58
Hope	MBSR	21	3.27	0.58	18	3.61	0.53	18	3.55	0.56	17	3.55	0.56	18	3.62	0.68
	WL	21	3.36	0.66	16	3.30	0.65	16	3.34	0.57	16	3.17	0.71	16	3.28	0.57
Spirituality	MBSR	21	2.50	0.85	18	3.01	1.12	18	2.96	1.14	17	2.90	1.12	18	2.91	1.14
	WL	21	2.34	0.71	16	2.15	0.59	16	2.16	0.76	16	2.24	0.69	16	2.19	0.76

FU, follow-up test. *M*, mean; *SD*, standard deviation. *TOT-M*, total score of mindfulness. *Appreciation beauty*, appreciation of beauty and excellence. *MBSR*, mindfulness-based stress reduction; *WL*, waitlist control. *Pre*, right before the intervention; *Post*, 1 week after the intervention; *1 month*, *3 months*, *and 6 months*, one month, three months, and six months after the intervention

**Table 4:** Linear mixed effect model tests of mindfulness and character strengths by time and condition using completers’ and ITT dataset

		Completers’ dataset				ITT dataset				
Outcomes	Model effect	*β*	*df*	*t*	*p*	*β*	*df*	*t*	*p*	95% CI
TOT-M	Time1	.01	39.49	0.22	.829	.00	149.31	0.06	.955	− 0.09, 0.09
	Time2	.01	32.29	1.38	.178	.01	87.53	1.06	.294	− 0.01, 0.04
	Time1* MBSR	.20**	38.96	3.54	.001^a^	.21**	157.71	3.19	.002^a^	0.08, 0.33
	Time2* MBSR	− .01	32.20	− 0.75	.461	− .01	119.07	− 0.61	.545	− 0.05, 0.02
Creativity	Time1	.05	35.36	1.16	.254	.01	112.70	0.12	.908	− 0.13, 0.14
	Time2	.01	32.71	0.55	.585	.00	62.60	0.05	.961	− 0.04, 0.04
	Time1* MBSR	.06	34.88	1.05	.302	.10	141.76	1.07	.284	− 0.08, 0.27
	Time2* MBSR	− .01	32.64	− 0.71	.485	− .01	85.97	− 0.23	.820	− 0.06, 0.05
Curiosity	Time1	− .02	36.87	− 0.41	.684	− .04	155.57	− 0.80	.424	− 0.15, 0.06
	Time2	.00	32.12	− 0.44	.661	.00	73.75	− 0.32	.750	− 0.03, 0.02
	Time1* MBSR	.11	36.24	1.88	.069	.15*	166.23	2.05	.042	0.01, 0.30
	Time2* MBSR	.00	32.16	− 0.21	.835	.00	82.91	0.00	.997	− 0.04, 0.04
Open-mindedness	Time1	.00	40.00	0.05	.957	.00	123.41	0.08	.938	− 0.10, 0.10
	Time2	.01	77.40	0.90	.372	.01	67.59	0.42	.675	− 0.02, 0.03
	Time1* MBSR	.07	39.15	1.26	.216	.09	131.71	1.27	.207	− 0.05, 0.23
	Time2* MBSR	.00	77.12	− 0.26	.794	.00	82.69	− 0.02	.987	− 0.04, 0.04
Love of learning	Time1	.00	32.48	− 0.01	.993	− .05	128.59	− 0.85	.399	− 0.17, 0.07
	Time2	.00	33.00	− 0.43	.667	.00	73.02	− 0.13	.898	− 0.03, 0.03
	Time1* MBSR	.05	32.33	0.99	.330	.10	134.61	1.26	.210	− 0.06, 0.27
	Time2* MBSR	.00	32.71	0.26	.796	.00	80.87	0.05	.964	− 0.04, 0.04
Perspective	Time1	− .01	33.69	− 0.39	.698	− .03	148.14	− 0.72	.474	− 0.12, 0.06
	Time2	.01	32.01	0.96	.344	.01	86.40	0.64	.526	− 0.02, 0.03
	Time1* MBSR	.10	33.36	2.00	.054	.12	145.67	1.92	.057	0.00, 0.25
	Time2* MBSR	− .01	32.06	− 0.46	.651	− .01	79.82	− 0.31	.760	− 0.04, 0.03
VIA-IS										
Bravery	Time1	.01	34.92	0.21	.835	− .01	105.62	− 0.18	.856	− 0.11, 0.09
	Time2	.01	32.14	1.54	.134	.01	71.11	0.81	.420	− 0.02, 0.04
	Time1* MBSR	.12*	34.58	2.64	.012	.11	138.30	1.69	.093	− 0.02, 0.25
	Time2* MBSR	.00	32.16	− 0.36	.720	.00	93.63	− 0.19	.851	− 0.04, 0.04
Perseverance	Time1	.01	35.70	0.27	.793	− .01	123.61	− 0.21	.837	− 0.11, 0.09
	Time2	.02	32.39	1.67	.105	.02	87.09	1.02	.311	− 0.02, 0.05
	Time1* MBSR	.07	35.39	1.34	.189	.11	139.25	1.52	.131	− 0.03, 0.24
	Time2* MBSR	− .03	32.43	− 1.94	.061	− .02	92.00	− 1.07	.286	− 0.07, 0.02
Zest	Time1	− .02	33.70	− 0.42	.674	− .08	149.89	− 1.34	.181	− 0.20, 0.04
	Time2	.01	32.00	0.84	.407	.01	99.52	0.51	.612	− 0.02, 0.04
	Time1* MBSR	.17*	33.47	2.65	.012	.24**	158.08	2.88	.004	0.07, 0.40
	Time2* MBSR	− .02	32.03	− 1.20	.239	− .02	104.45	− 0.66	.512	− 0.06, 0.03
Love	Time1	− .07	66.04	− 1.97	.053	− .08	123.61	− 1.63	.106	− 0.19, 0.02
	Time2	.03**	44.72	2.73	.009	.02	78.00	1.25	.217	− 0.01, 0.05
	Time1* MBSR	.19***	64.53	3.91	< .001^a^	.19**	138.62	2.74	.007	0.05, 0.33
	Time2* MBSR	− .03*	44.78	− 2.23	.031	− .02	101.32	− 0.99	.323	− 0.06, 0.02
Social intelligence	Time1	.01	35.01	0.41	.688	.01	128.46	0.14	.887	− 0.07, 0.09
	Time2	.01	32.00	1.64	.111	.01	90.74	0.78	.436	− 0.01, 0.03
	Time1* MBSR	.03	34.79	0.60	.553	.05	130.34	0.81	.421	− 0.07, 0.16
	Time2* MBSR	− .01	32.05	− 0.63	.531	.00	82.80	− 0.20	.846	− 0.04, 0.03
Forgiveness	Time1	.00	37.40	0.09	.930	− .01	160.40	− 0.21	.835	− 0.12, 0.09
	Time2	.02	67.37	1.91	.061	.02	94.08	1.55	.126	− 0.01, 0.05
	Time1* MBSR	.09	36.76	1.38	.176	.10	163.30	1.27	.205	− 0.05, 0.24
	Time2* MBSR	− .01	65.77	− 1.01	.318	− .01	100.05	− 0.75	.454	− 0.05, 0.02
Self-regulation	Time1	.03	34.35	0.64	.529	.00	148.46	0.06	.949	− 0.11, 0.12
	Time2	.01	32.02	0.85	.402	.01	89.67	0.60	.549	− 0.02, 0.04
	Time1* MBSR	.09	33.63	1.47	.151	.12	161.50	1.49	.137	− 0.04, 0.27
	Time2* MBSR	− .01	32.05	− 0.57	.576	− .01	107.77	− 0.35	.730	− 0.04, 0.03
Appreciation beauty	Time1	− .08*	46.86	− 2.27	.028	− .09	127.27	− 1.61	.109	− 0.21, 0.02
	Time2	.02*	86.07	2.13	.036	.02	80.59	1.00	.319	− 0.02, 0.05
	Time1* MBSR	.21***	46.35	4.20	< .001^a^	.22**	144.86	2.84	.005	0.07, 0.38
	Time2* MBSR	− .02	85.93	− 1.80	.075	− .02	97.17	− 0.76	.451	− 0.06, 0.03
Gratitude	Time1	− .09	43.47	− 2.74	.009	− .12*	93.68	− 1.99	.049	− 0.23, 0.00
	Time2	.02	52.17	2.14	.037	.01	55.91	0.71	.483	− 0.02, 0.05
	Time1* MBSR	.23***	42.72	5.13	< .001^a^	.27***	105.12	3.42	.001^a^	0.11, 0.43
	Time2* MBSR	− .03*	51.34	− 2.40	.020	− .02	72.38	− 0.93	.354	− 0.07, 0.02
Hope	Time1	.03	34.68	0.57	.571	− .02	120.41	− 0.31	.754	− 0.14, 0.10
	Time2	.00	33.05	− 0.34	.740	− .01	70.53	− 0.52	.606	− 0.05, 0.03
	Time1* MBSR	.13	34.36	1.88	.069	.17*	150.87	2.07	.041	0.01, 0.33
	Time2* MBSR	.01	32.95	0.39	.699	.01	105.26	0.50	.620	− 0.03, 0.06
Spirituality	Time1	− .04	32.01	− 0.77	.447	− .08	124.34	− 1.03	.304	− 0.23, 0.07
	Time2	.01	32.23	0.57	.570	.00	84.61	0.11	.915	− 0.04, 0.05
	Time1* MBSR	.26***	32.01	3.51	.001^a^	.32**	140.48	3.09	.002	0.12, 0.53
	Time2* MBSR	− .02	32.24	− 1.14	.263	− .01	97.79	− 0.47	.642	− 0.08, 0.05

*TOT-M*, total score of mindfulness. *Appreciation beauty*, appreciation of beauty and excellence. *MBSR*, mindfulness-based stress reduction. *ITT*, intent-to-treat. *95% CI*, 95% confidence interval. *훽*, standardized linear regression coefficients. *df*, degree of freedom. Negative coefficients indicate that participants in the intervention condition had a greater decrease over the specific time period compared with waitlist control participants. Positive coefficients indicate that participants in the intervention condition had greater gains over the specific time period compared with waitlist control participants

*p < .05; **p < .01; ***p < .001. ^a^Results with the superscript indicate statistical significance using the Bonferroni corrections (*p* < .0019) for multiple comparisons (27 tests, which also takes the models that were computed within the same project but not reported in the current manuscript into consideration)

There was a significant increase (after Bonferroni corrections) in the total score of mindfulness, indicating that the MBSR training was effective in enhancing participants’ dispositional mindfulness. Of the proposed list of character strengths that were considered to overlap with mindfulness, the following character strengths showed significant condition effects from pre-test to post-test (i.e., when evaluated by examining the Time1*Condition interaction). Compared with the WL, participants in the MBSR condition showed significant increases in love (*β* = .19, *p* < .001), appreciation of beauty (*β* = .21, *p* < .001), gratitude (*β* = .23, *p* < .001), and spirituality (*β* = .26, *p* < .001). They also showed a trend toward significant increases in zest (*β* = .17, *p* < .05) and bravery (*β* = .12, *p* < .05), when taking multiple comparisons into consideration. The results for the strength—appreciation of beauty—warrant caution due to the fact that the WL showed a decreasing trend over the same period. In contrast, the following character strengths did not show significant condition effects (after Bonferroni corrections): creativity, curiosity, open-mindedness, love of learning, perspective, perseverance, social intelligence, forgiveness, self-regulation, and hope, indicating that those strengths were not changed after an eight-week MBSR training.

After the mindfulness training, the majority of the strengths that were enhanced had not declined six months after the intervention. The exceptions were love and gratitude, which showed a trend toward slight decreases at the follow-up tests. The declining trend of these two strengths for the MBSR group over Time2 might be traced back to the increasing trend of the two strengths for the WL group over Time2. The results of the intention-to-treat analyses using the MI dataset showed a similar pattern. The effects were less statistically significant in some models based on imputed data, which is probably due to anomalies produced by MI when dealing with skewed data. All the estimates obtained from the completers’ datasets fell within the 95% confidence intervals of the imputed estimates, which showed that comparable results would have been obtained if there had been no dropouts over time.

## Discussion

**Fig. 3:**
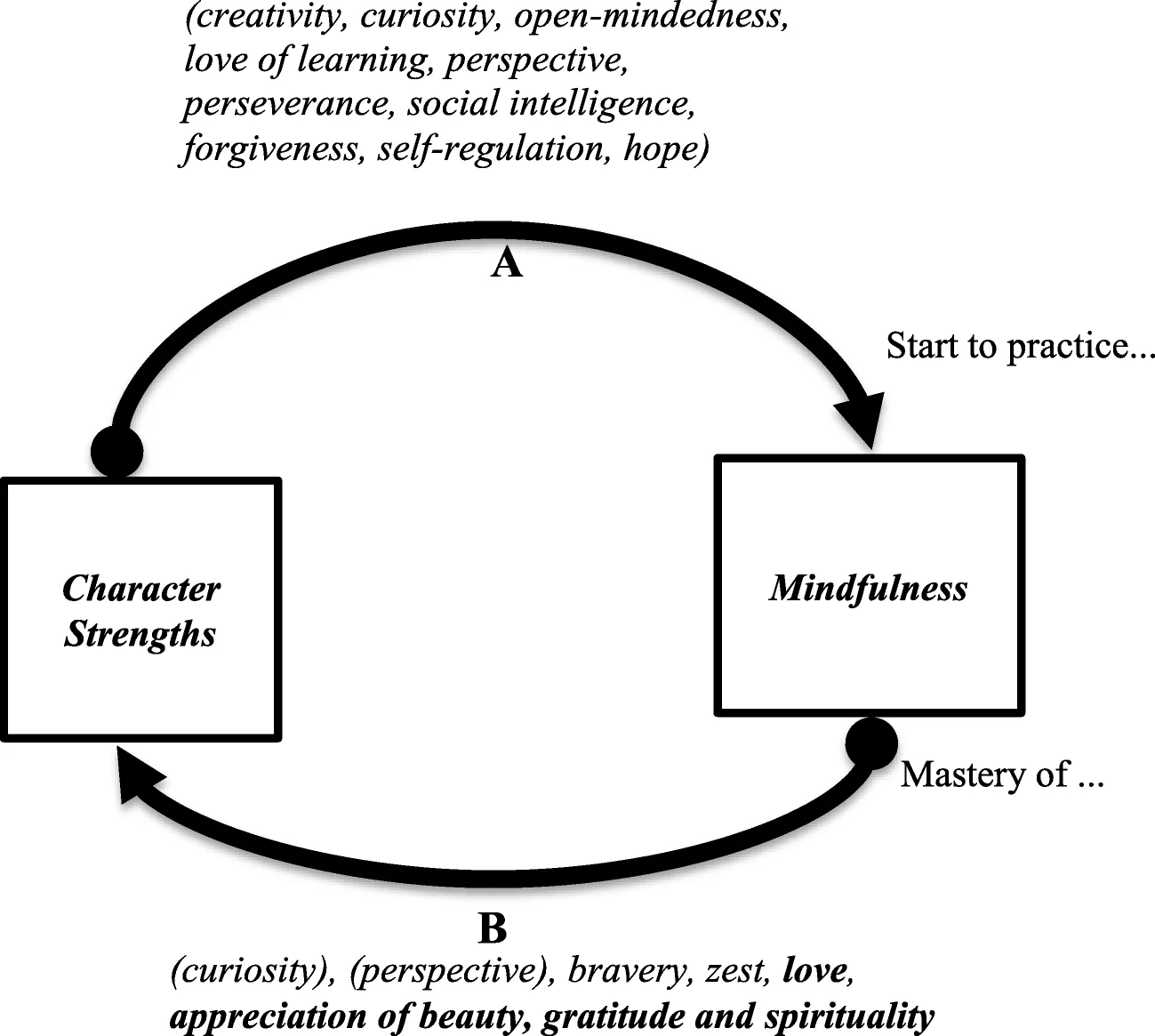
The revised mutual support model of mindfulness and character strengths. Path B, the strengths in **bold** indicate statistical significance after the Bonferroni corrections (*p* < .0019). The strengths not in bold indicate a less statistical power (*p* < .05), while strengths in parentheses indicate an even smaller statistical power (*p* < .10). Path A, the strengths presented in path A could not be tested directly in the present study

Based on these results, an updated and more detailed model of mutual support can be generated. The revised mutual support model is shown in Fig. 3. The strengths that were indeed increased after the mindfulness training were love, appreciation of beauty, gratitude, and spirituality, which should be in path B of the model. The strengths of bravery and zest should still be considered for path B of the model even though they have less statistical power (*p* < .05). The effects for curiosity and perspective are smaller (*β* = .11 and .10) and with even weaker statistical power (*p* < .10). These strengths were assumed to contribute to both path A and path B, because if they were strengths that motivate people to start mindfulness practice, they would have less room for improvement. Those strengths are included in parentheses in Fig. 3 to indicate that further verification is required. Although not tested directly in the present study, the remaining strengths were considered to overlap with mindfulness contribute to path A of the model. These were creativity, open-mindedness, love of learning, perseverance, social intelligence, forgiveness, self-regulation, and hope.

## General Discussion

This study presents preliminary evidence of relationships between mindfulness and character strengths within the VIA classification framework (Peterson and Seligman 2004). Meaningful relationships were observed between the two constructs, and the findings provide initial evidence for the mutual support model of mindfulness and character strengths. The results extend existing findings (Duan 2016; Duan and Ho 2018), as a more comprehensive measurement of character strengths was utilized to capture the full picture of the interconnections with mindfulness and its facets. In addition, the randomized-control design offers initial evidence that certain character strengths can indeed be fostered by a mindfulness training.

Based on these findings, links between mindfulness and character strengths can be established and a mutual support model that represents those links is proposed: certain character strengths facilitate the practice of mastering mindfulness, while the mastery of mindfulness enhances certain strengths. Both are seen as malleable in that they can be cultivated and developed with deliberate processes. It is clear from a conceptual standpoint and based on empirical findings that mindfulness seems to exert an influence on the development of certain character strengths, notably curiosity, perspective, bravery, zest, love, appreciation of beauty, gratitude, and spirituality. Conversely, from a conceptual viewpoint, it also makes sense that certain character strengths have some sort of influence on mindfulness, such as facilitating its occurrence or enriching the practice. Those character strengths are creativity, curiosity, and perspective. Therefore, it can be boldly assumed that this mutual support could work in a sort of cyclical fashion: through practice, mindfulness is enhanced, and this, in turn, increases the relevant character strengths. Some of these improved character strengths might presumably then feedback into improving the quality of mindfulness practice which then enhances mindfulness and so on in a continuous cycle. Through enabling increased awareness of ourselves, mindfulness allows us to develop our character strengths to a greater extent; in return, increased character strengths (such as self-regulation and curiosity) improve our ability to better pay attention and explore the present moment (Christopher and Colgan 2014).

The results of follow-up tests collected up to six months after the mindfulness training also suggest that the enhancement of specific character strengths does not decline over a longer period following training. The main reason for these lasting effects seems to be the regular home practice of the participants in study 2, who completed homework on a regular basis during and after the eight-week course.

The most robust correlations between mindfulness and character strengths were identified as hope, bravery, curiosity, social intelligence, zest, love, perspective, and gratitude. These happen to include the strengths that correlated most with life satisfaction across different samples (i.e., hope, zest, gratitude, love, and curiosity; e.g., Brdar and Kashdan 2010; Buschor et al. 2013; Ruch et al. 2007, 2010). This suggests that mindfulness and character strengths could be two different but connected pathways that lead to well-being. Is mindfulness training actually a direct training in character strengths that are related to life satisfaction, and thus a pathway to improve well-being? Future studies could investigate the specific role of those life satisfaction–related strengths in this process by testing their mediational role in the influence of mindfulness training on well-being.

## Limitations and Future Research

Several limitations of the present study warrant mention and indicate that the results should be interpreted with caution. First, study 1 relied exclusively on self-reported data gathered online from participants via the Internet. Thus, a selection bias is to be expected because the participants are more likely to be people who are interested in positive psychology in general or are curious about self-discovery. This bias was minimized by advertising the study on a broad basis and by addressing the importance of the study to the targeted participants through invitation letters and e-mails. Second, although study 2 was balanced with respect to demographics and outliers were checked before the analysis, the sample size was small. Therefore, some of the non-significant results for specific character strengths might be due to the small sample size; hence, the possibility arises that these effects remain undetected. Other problems associated with small sample sizes may also apply, including low statistical power and capitalization on chance, so cross-validation using a larger sample remains desirable.

Third, in study 2, the FFMQ was always completed before VIA-IS, which might have produced possible order effects in answering the instruments. For example, it is possible that answering mindfulness questions first can prime participants in a way that could activate their specific character strengths (e.g., appreciation of beauty). Fourth, study 2 did not use an active control group. This leaves open the possibility that demand characteristics and/or placebo effects may have played a role in the results. Fifth, the strengths presented in path A of the mutual support model were not examined because it could only be partially tested with our current sample. Only those character strengths that facilitate mindfulness training might be identified through the analysis (e.g., by using pre-tests of character strengths score to predict the improvement of mindfulness score). However, which character strengths potentially lead them to start practicing mindfulness training would still be not clear. An additional sample is needed, who are similar in age, gender, and education, but have no interest in mindfulness training at all. Sixth, the mindfulness training was only an eight-week course. Although this sufficed to enable changes in specific character strengths, it is still a very short period compared with long-term practitioners of mindfulness. This should be taken into consideration when interpreting the results, as it is possible that some character strengths need a longer duration to improve. Future research could employ longitudinal designs with follow-up tests of longer intervals and include participants who continue to practice over a longer period of time. Seventh, the particular training program of MBSR contains a variety of modules and exercises, making it impossible to determine the specific elements responsible for the observed changes in character strengths. The elements include, for example, breathing exercises, sitting together, and doing yoga, and any of these or a combination could be responsible for the observed changes. Future research could segment the elements of MBSR to clarify which lead to specific changes in character strengths.

## Data Availability

All data are available at the Open Science Framework (https://osf.io/dgf32/).
